# No protection of permafrost due to desertification on the Qinghai–Tibet Plateau

**DOI:** 10.1038/s41598-017-01787-0

**Published:** 2017-05-08

**Authors:** Qingbai Wu, Wenbing Yu, Huijun Jin

**Affiliations:** State Key Laboratory of Frozen Soils Engineering, Northwest Institute of Eco-Environment and Resource, Chinese Academy of Science, Lanzhou, 730000 China

## Abstract

Desertification of tundra regions may form an escalating cycle with permafrost degradation where more permafrost thaw leads to continued desertification. This traditional viewpoint has been challenged in recent reports that state desertification protects the underlying permafrost. However, our measurements of soil temperature from nine sites in the Honglianghe River Basin, interior Qinghai-Tibet Plateau, show that desertification can degrade permafrost. If one compares the permafrost temperatures at sites with thin sand covers (e.g. site Yu-7, permafrost temperature of −0.64 °C; site Yu-6, permafrost temperature of −1.15 °C) with that of site Xie-1 (−0.65 °C, with a 120-cm-thick sand cover), the permafrost temperature is not significantly different. It is clear that a thick sand cover does not influence the underlying permafrost temperature. Our observations support traditional geocryological knowledge which states that, under most circumstances, desertification does not protect, but rather degrades, permafrost.

## Introduction

Permafrost is defined as ground that remains continuously at or below 0 °C for at least two consecutive years^1^. Permafrost is a product of long-term energy exchange between the ground surface and atmosphere and is therefore a condition of ground climate^2^. The difference in the macro-scale distribution of permafrost is controlled by the climate^3^; however, the difference in the local conditions, such as the topography, vegetation, snow cover, soil and geological conditions, can significantly modify the thermal impacts of the climate^4–6^ and result in local-scale anomalies in permafrost distribution^3^.

One of the local-scale factors influencing permafrost distribution is dominantly surface cover, e.g. vegetation, snow cover, and water^7^. Many studies have presented the thermal effects of local-scale surface cover on the active layer processes, e.g., the hydrothermal regimes and ground freezing-thawing in the active layer, active layer thickness, and thermal states of permafrost^3, 8–11^. However, little focus has been placed on the hydrothermal impacts of desertification (sand layer or dune formation over the frozen ground) on the thermal regimes of soils in the active layer and the underlying permafrost.

Permafrost underlies approximately 70% of the land area of the Qinghai-Tibet Plateau (QTP), the highest and most extensive plateau permafrost on Earth^12–14^. Observational evidence demonstrates that warming, thawing and degrading of the plateau permafrost has occurred during the past few decades^11, 13, 15–19^. The effect of permafrost degradation in the Qinghai-Tibet Plateau dries the ground surfaces because the active layer usually thickens, the permafrost table lowers and vegetation becomes more fragmented and sparse. The result is an increase in wind action, enhanced deflation and Aeolian deposition, and increased potential risk of desertification^16, 20–22^.

Since the 1960s, grassland deterioration and desertification have occurred in some parts of the QTP^23–25^ and Northeast China^25^. However, a number of recent studies^26–28^ have concluded that a surface cover of wind-blown sand actually protects the underlying permafrost on the QTP. These studies are based on short-term data from only one field site and are in conflict with traditional geocryological knowledge^20, 21, 29, 30^. Accordingly, we review the relationships between land desertification and permafrost degradation at the Qinghai-Tibet Plateau and discusses the spatial distribution of permafrost on the QTP. We assesses the effect of a surface sand layer on ground heat transfer via modeling informed by field and laboratory measurements.

## Data and Method

**Figure 1:**
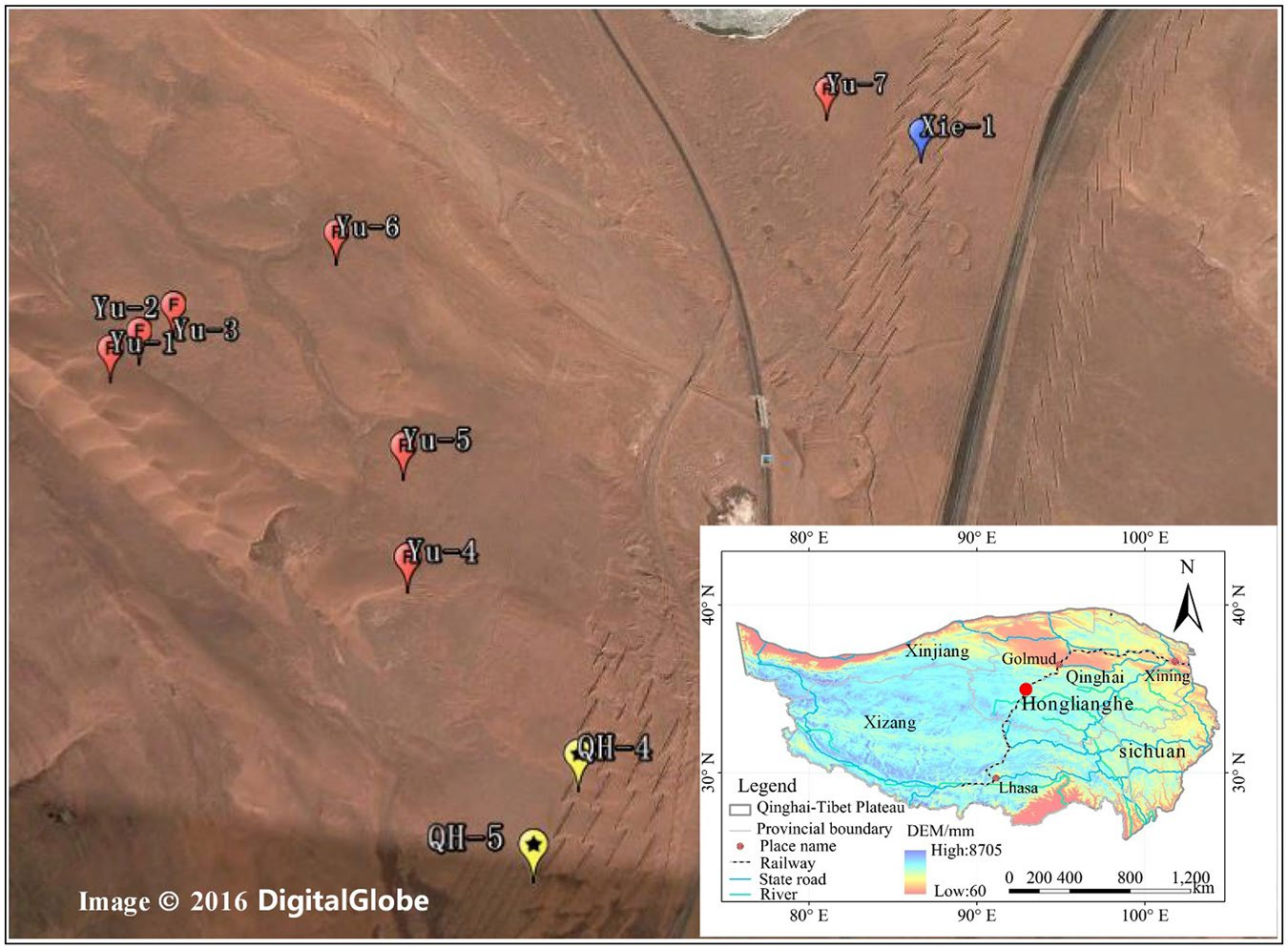
Map of observation sites at the Honglianghe River Basin along the Qinghai-Tibet Highway of the interior Qinghai-Tibet Plateau (the map was edited and generated using Google Earth, Image© 2016 DigitalGlobe; the small map was edited and generated using ArcGIS 8.0, which is American GIS Software (http://www.esrichina.com.cn/softwareproduct/ArcGIS/); Qingbai Wu created this map).

Nine sites around the Xie *et al*.^28^ study site were established in 2012 for ground temperature monitoring in the Honglianghe River basin (Fig. 1; Table 1). The geographical information, sand cover, permafrost temperature and active layer thickness are shown in Table 1. These sites had different thicknesses of sand cover. One site (site Yu-7) is located near to the soil temperature monitoring site discussed by Xie *et al*. (site Xie-1)^28^ (see Fig. 1).

**Table 1:** Geographic information, surface conditions, mean annual ground temperature (MAGT), active layer thickness (ALT), seasonally frozen ground thickness (SFT) and observation methods for ground temperatures (GT) at the nine study sites.

Sites	Longitude (°E)	Latitude (°N)	Surface condition	MAGT (°C)	ALT (m)	SFT (m)	Observation methods for GT
Yu-1	35.0490	93.0069	Sand cover, 3.3 m in thickness	1.03		—	Manual
Yu-2	35.0495	93.0071	Desert with sparse vegetation	0.58		—	
Yu-3	35.0498	93.0076	Desert with sparse vegetation	0.89		—	
Yu-4	35.0466	93.0119	Sand cover, 3.0 m in thickness	0.84		4.0	Automatically collected by data logger CR3000
Yu-5	35.0481	93.0115	Desert with sparse vegetation	0.92		3.8	
Yu-6	35.0513	93.0097	Desert with sparse vegetation	−1.15	2.0		Manual
Yu-7	35.0542	93.0147	Desert with sparse vegetation	−0.64	3.5		
QH-4	35.0444	93.0147	Sand cover, 30 cm in thickness	0.30		4.5	Automatically collected by data logger CR3000
QH-5	35.0434	93.0143	Sand cover, 115 cm in thickness	−0.74	3.2		

In this area of the QTP, climate data from Wudaoliang (from 2010 to 2014, 15 km from the study sites) indicates mean annual air temperature varies from −3.8 °C (2010) to −4.6 °C (2012) with an average of −4.2 °C. Annual precipitation varies from 273 mm (2013) to 425 mm (2012) with an average of 369 mm. Mean annual air temperatures in the summer months (JJA) vary from 4.13 to 7.2 °C, with an average of 5.7 °C. In the winter months (DJF) mean annual air temperatures vary from −13.47 to −14.67 °C, with an average of −14.0 °C.

Soil temperature were measured at depths of 0.1 to 18.0 m using thermistor strings at depth increments of 0.5 m that were assembled by the State Key Laboratory of Frozen Soils Engineering (SKLFSE) at Lanzhou, China. The temperature accuracy of these sensors is ±0.05 °C. For all sites, the *in-situ* measurements were conducted in two ways: (i) some were recorded manually on the 5th and 20th days of each month, (ii) others were collected daily by data loggers (CR3000, Campbell Scientific Inc., USA). Three sites were underlain by permafrost (Yu-6, Yu-7, QH-5), the others experience deep seasonal frost.

## Results

### Subsurface soil temperatures

**Figure 2:**
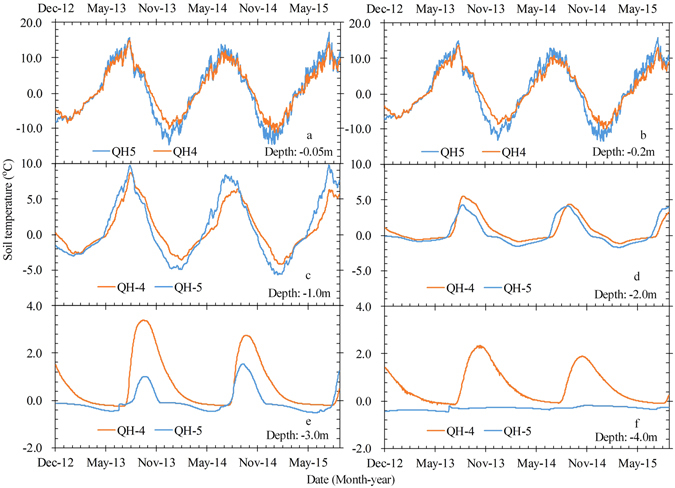
Variations in soil temperatures at depths of 0.05 m (**a**), 0.2 m (**b**), 1.0 m (**c**), 2.0 m (**d**), 3.0 m (**e**), and 4.0 m (**f**) at QH-4 (30 cm sand layer) and QH-5 (115 cm sand layer) in the Honglianghe River Basin during the observation period from 1 December 2012 to 28 August 2015.

**Figure 3:**
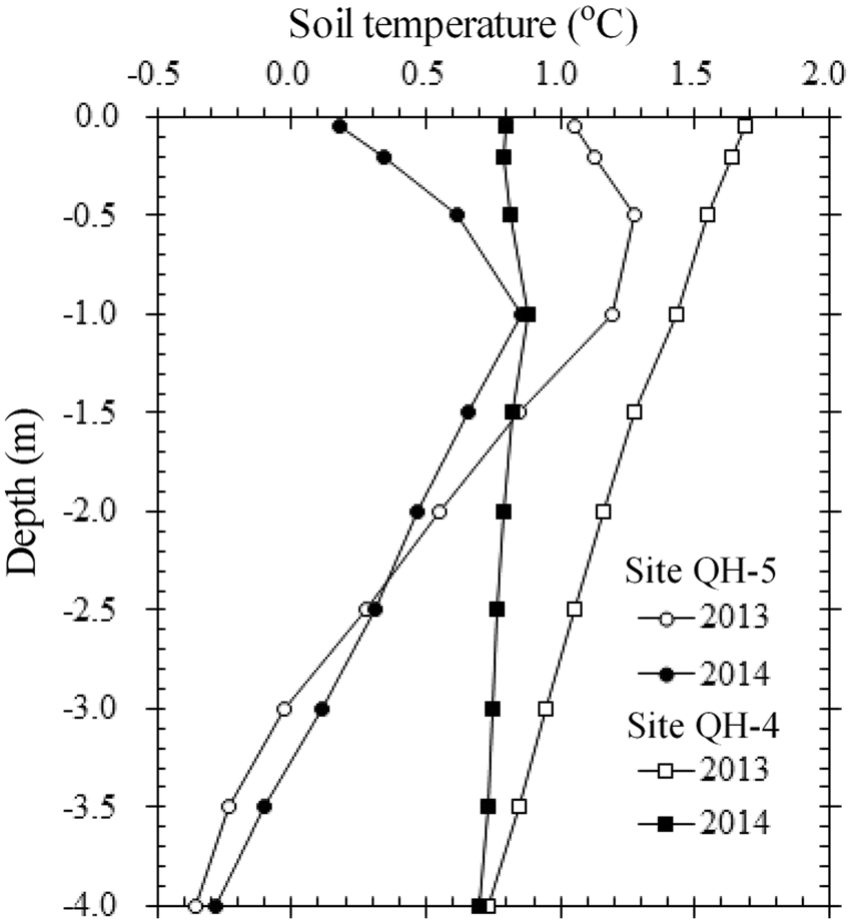
Mean annual soil temperature in 2013 and 2014 at 0.1 to 4.0 m depths at sites QH-4 (30 cm sand layer) and QH-5 (115 cm sand layer) in the Honglianghe River Basin.

Figure 2 shows variations in soil temperatures at depths of 0.05 m to 4.0 m under (i) a 0.3-m-thick sand cover at site QH-4 (deep seasonal frost) and (ii) a 1.15-m-thick sand cover at site QH-5 (permafrost). Ground temperatures at site QH-4 are lower than those at site QH-5 during the summer months but higher during the winter months in the near-surface (0.05 m to 2.0 m depth) (Fig. 2a–d). However, at depths of 3 to 4 m, temperatures at QH-5 are colder than those at QH-4 (Fig. 2e,f) and the MAGT at site QH-5 is lower than that at site QH-4 (Fig. 3). It is clear that the mean daily and mean annual ground temperatures change with the thickness of the sand cover; as the thickness of the sand cover increases, the soil temperature decreases. This pattern is similar to the pattern observed by Xie *et al*.^28^ but it does not explain how the sand layer (or sand veneer) protects the underlying permafrost from thermal change. In other words, conclusions cannot be drawn from ground temperatures observed at just one borehole.

### Thermal regime of permafrost under sand cover

**Figure 4:**
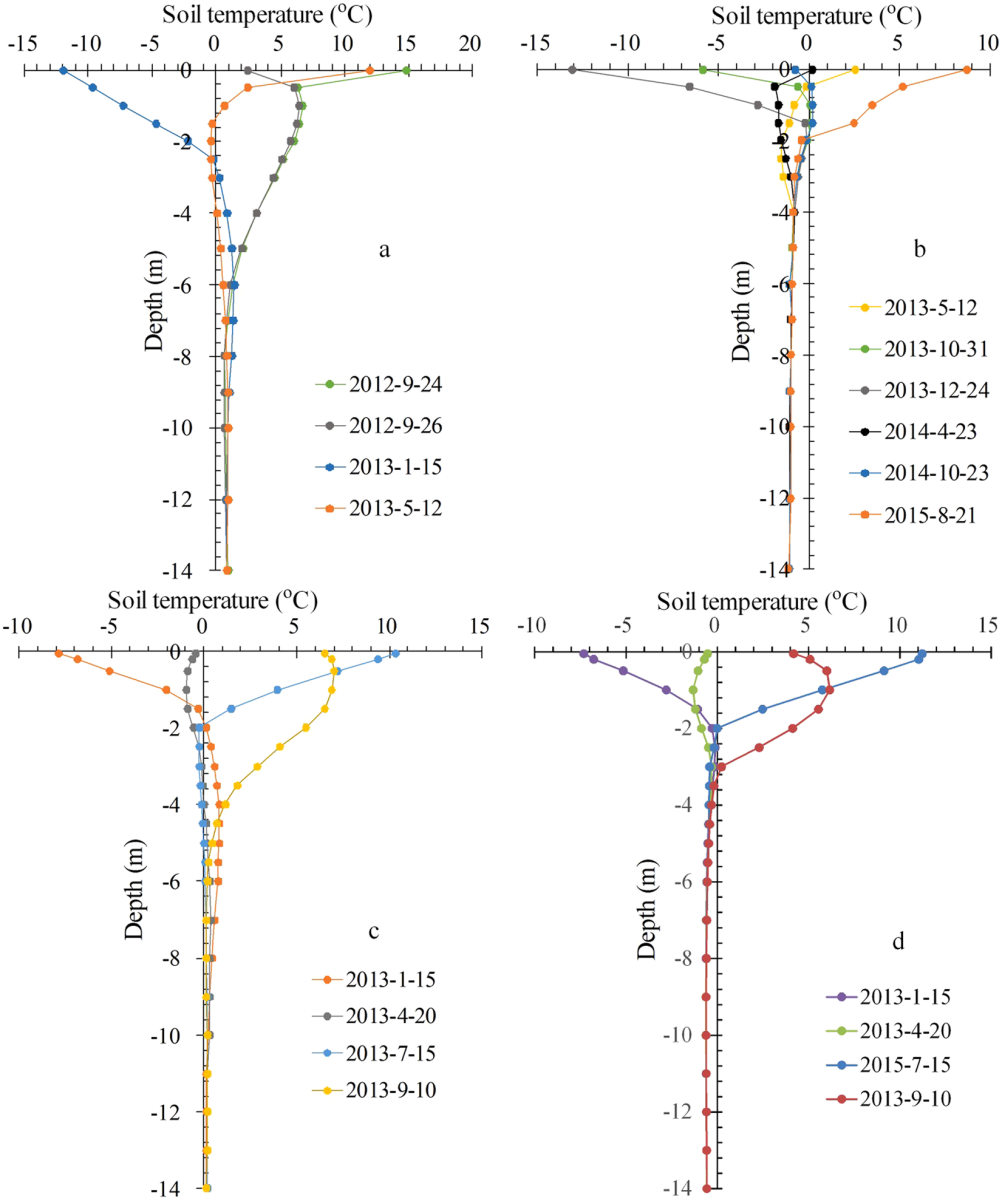
Soil temperature profiles at sites with (**a**) a 3.0-m-thick sand cover (Yu-4), (**b**) desertified land with sparse vegetation (Yu-6), (**c**) a 30-cm-thick sand cover (QH-4), and 1.15-m-thick sand cover (QH-5).

Figure 4 shows the thermal regimes of ground under different sand covers that were measured at observation sites close to the observation site of Xie *et al*.^28^ in the Hongliang River Basin. The data demonstrates that seasonal frozen ground can be found under a 3.0-m-thick sand cover at site Yu-4 and also under a 0.3-m-thick sand cover at site QH-4. At the same time, the permafrost temperature under a sand dune with sparse vegetation at a depth of 14 m is −1.15 °C (site Yu-6) yet it is −0.74 °C under a 1.15-m-thick sand cover at site QH-5. When one compares the permafrost temperature at dune-free sites (site Yu-7, −0.64 °C and site Yu-6, −1.15 °C) with that at site Xie-1 (with a 120-cm-thick sand cover), the permafrost temperature at site Xie-1 is −0.65 °C^28^, clear, therefore, that a thick sand cover does not significantly influence the underlying permafrost temperature.

### Permafrost temperature

The permafrost at the nine sites monitored by the authors shows a strong spatial heterogeneity. Regarding MAGT, permafrost has strong spatial heterogeneity caused by various thicknesses of the sand cover in the Honglianghe River area. For example, no permafrost is found at sites Yu-1 to Yu-5 (desert with sparse vegetation) in spite of variations in sand-cover thickness. Permafrost also occurs at sites Yu-6 and Yu-7 (deserts with sparse vegetation) and at site QH-5 (with 115-cm-thick sand cover). The mean annual ground temperature (MAGT) at two non-permafrost sites ranges from +0.3 °C at the YU-6 site to −1.15 °C at the QH-4 site. The average is −0.6 °C. The MAGTs from three Xie *et al*.^28^ sites are estimated from Fig. 1 to range from approximately −0.5 °C to −0.7 °C.

If sand cover, or desertification, has a role in protecting the underlying permafrost, it should be observed widely across the Honglianghe River Basin. However, our observations show permafrost does not exist at sites with either a sand cover of 3.0 to 3.3 m in thickness or with barren surfaces possessing very sparse vegetation. On the other hand, permafrost occurs at sites with possess either a thin sand cover (30 cm to 115 cm in thickness) or are barren and with sparse vegetation or barren (Table 1). Obviously, spatial difference alone cannot verify whether sand cover, or desertification, protects the underlying permafrost.

## Discussion

### Relationships between desertification and permafrost

Permafrost plays a fundamental role in the alpine ecosystems of the Qinghai-Tibet Plateau^31, 32^. If warming is not accompanied by increased precipitation, permafrost degradation causes a lowering of the water table, vegetation degradation and desertification^13, 21, 29, 30, 32, 33^. A positive feedback mechanism is also present: the decreased vegetative coverage increases surface energy fluxes that can lead to ground warming and further desertification^32^.

Interestingly, if desertification can protect underlying permafrost, as claimed by Xie *et al*.^26–28^, it follows that Late Pleistocene permafrost should be well-preserved in the cold deserts of western China. The maximum southern limit of latitudinal permafrost in China during the Last Glacial Maximum (LGM) was approximately 5–10° latitude beyond the present-day southern permafrost limit at 46°–47°N^34, 35^. However, no sand-wedge casts or ice-wedge pseudomorphs or other periglacial phenomena indicative of the occurrence of either past or present permafrost have been found on or around sand dunes in the desert regions of western China^34^; however, they have been identified in oases such as wetlands, river valleys and terraces in those desert regions. Actually, in many high plateau and alpine regions, permafrost is generally absent in deserts, although talik is extensively identified under deserts or desertified lands with active sand dunes.

### Local heterogeneity of permafrost occurrence

The permafrost distribution across the Qinghai-Tibet Plateau has a strong spatial heterogeneity that is influenced by local factors such as vegetation, soil texture and stratigraphy. Illustrations of this heterogeneity include (i) the distribution of permafrost in the Liangdaohe River Basin along the QTH^36^, (ii) the distribution of permafrost along the eastern side of the QTH where permafrost occurs extensively under paludal alpine meadows with a mean annual ground temperature (MAGT) of approximately −1.2 °C and a permafrost thickness of more than 60 m^37^, no permafrost can be found along the western side of the highway due to human-induced degradation of the alpine meadows, and (iii) the distribution of permafrost in the Beiluhe River Basin along the QTR where the MAGT ranges from −0.4 °C to −1.8 °C across an area of 5 km^2, 38^ and where, at the Fenghuoshan Mountain Pass, the MAGT on the north and south slopes differs by approximately 2 °C, and at the Kunlunshan Mountain Pass by approximately 1.5 °C^39^. Thus, permafrost distribution with local heterogeneity in the Honglianghe River basin merely reflects the distribution of permafrost observed elsewhere on the QTP.

### The influence of heat and moisture

Xie *et al*.^28^ suggest that a sand cover or a non-vegetated surface affects the surface radiation and energy budgets of the ground surface as well as the heat and moisture exchange during ground freezing and thawing^40^. For example, the soil temperature under a sand cover with a thickness of less than 20 cm is typically colder than that without a sand cover, but the soil temperature under a sand cover with a thickness greater than 20 cm is typically warmer than that without a sand cover^30, 41, 42^. These conclusions are the result of a short-term studies that fails to address the reasons why a sand cover affects the underlying permafrost.

Physical modelling provides some perspective to this problem. According to long-term permafrost temperatures observed in the Beiluhe Basin, the rate of the increase of temperature under alpine meadows is larger than that under alpine steppes and barren lands and is smallest under desert grasslands^11^. Such variations in permafrost temperatures occur because the thaw of warm permafrost (MAGT at −0.17 °C) requires a large amount of latent heat that slowly attenuates the variation of permafrost temperature^43^.

Although details of the heat-moisture transfer that occurs in the surface sand layer are largely unknown, the Fourier Law can be used to approximate the heat fluxes involved using known ground temperatures at a given range of depths:

1 $$q=-\lambda \frac{{T}_{2}-{T}_{1}}{\Delta z}$$

where *λ* is the thermal conductivity of soil (W.m^−1^°C^−1^). *T*_1_ and *T*_2_ are the temperatures of the lower and upper surfaces of the soil layer (°C), respectively, and *Δz* is the thickness of the soil layer (m). The thermal conductivity of soil is a function of bulk density, moisture (mainly water or ice) content and temperature gradient^44^. Therefore, the soil moisture content determines the heat fluxes at a given depth. The thermal conductivity of sand in the Honglianghe River Basin, as measured in a laboratory, ranges from 0.85 to 2.75 W/(m°C) in accordance with an increase in the water content from 3% to 15%^45^. The thermal conductivity of dry sand is 10 times less than that of water-saturated sand.

**Figure 5:**
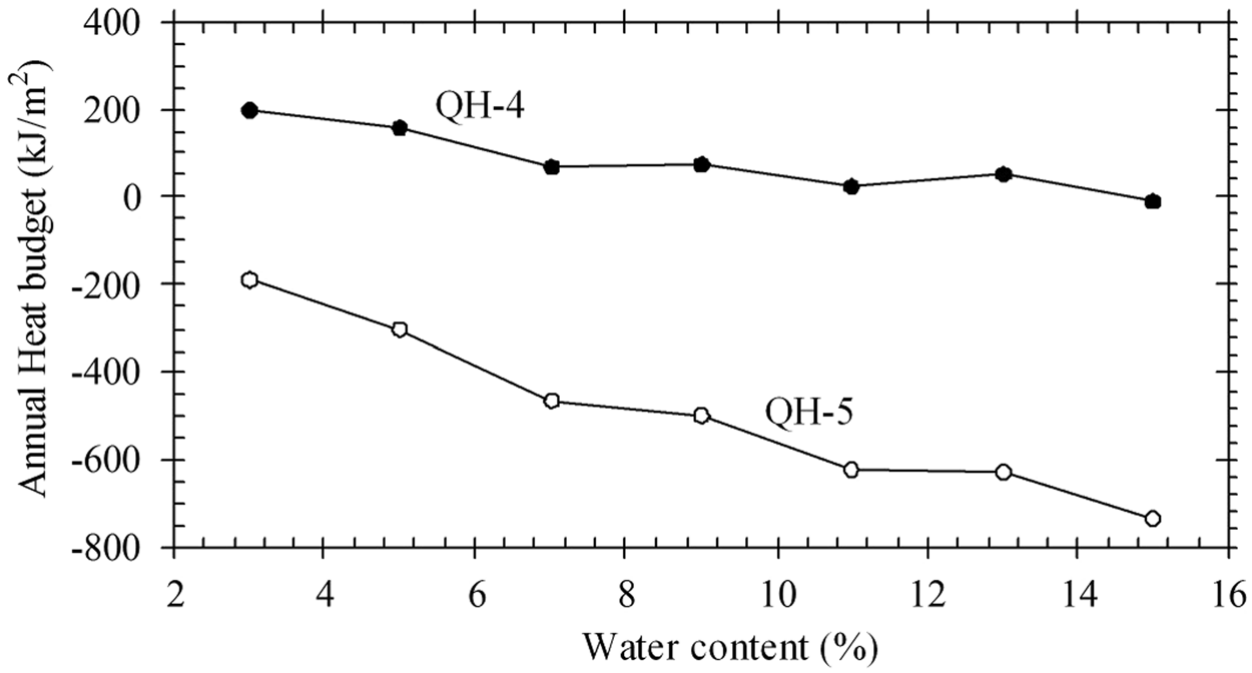
Calculated annual budget of the heat entering the sand layer at depths from 5 cm to 50 cm at sites QH-4 (30 cm sand layer) and QH-5 (115 cm sand layer).

To understand the thermal effect of soil moisture content on heat flux, one can use equation (1) and typical thermal conductivity values (Table 2) to calculate the heat fluxes entering the sand layer at depths from 5 cm to 50 cm. It is assumed that the moisture content of aeolian sand varies from 3% to 15%. By integrating the heat fluxes over a whole year, the annual budget of heat entering the sand layer at depths from 5 cm to 50 cm can be obtained. This is shown in Fig. 5.

**Table 2:** Laboratory-measured thermal conductivity of aeolian sand in the Honglianghe River Basin (Dry density: 1650 kg/m^3^).

Moisture content (%)		3	5	7	9	11	13	15
Thermal conductivity (W/(m^2^°C))	Frozen	1.27	1.54	1.86	2.01	2.28	2.38	2.57
	Thawed	1.35	1.5	1.62	1.75	1.88	2.01	2.06

It is clear that the annual heat budget of the sand layer progressively decreases with an increase in moisture content (Fig. 5). In other words, dry sand is in a state of extensive absorptive heat over the course of a year; this is a disadvantage to the protection of the underlying permafrost. For example, for site QH-5 (with permafrost), the annual heat budget in the active layer is 734 kJ/m^2^, but in the Chumaerhe Plain, where the climate condition is similar to that of the Honglianghe River region, the annual heat budget in the active layer at a depth of 50 cm to 1.0 m can be up to 12568 kJ/m^2^. It can be concluded, therefore, that a sand layer with high moisture content can better preserve the underlying permafrost only if heat convection is not considered.

Generally, high water content can be a double-edged sword. On the one hand, ice-rich permafrost is difficult to thaw because of latent heat; on the other hand, frequent water movement induces high amounts of heat convection. The latter is many times more effective than heat conduction, radiation, or their combination. In addition, soils with high water content are difficult to freeze because of latent heat. Therefore, discussion must focus upon permafrost that already contains a high ice content. In this situation, what is the role of the sand layer? According to Xie *et al*., it acts as a thermal diode, having better heat removal properties but being less effective with respect to heat absorption^28^. In other words, it serves similarly to a coarse block field, as applied successfully to QTR embankments^46^. Peat is another example. Soil moisture is typically low during summer but it becomes water-saturated and ice-rich in autumn and winter, at which time it has a much higher thermal conductivity. This is well documented in the geocryological literature. As a result, peat favours the preservation of marginal permafrost. Unfortunately, the sand layer does not have such unique thermal properties.

A further consideration is that an aeolian sand layer, or a sand dune, has a high infiltration rate and a very low water holding capacity, i.e., rainfall quickly infiltrates. Thus, it is difficult to maintain a high water content within a sand layer in a cold desert environment. For example, *in-situ* drilling in the Honglianghe River Basin showed that the water content within sand layers was always low. At site Yu-4 (see Table 1), the moisture content of the sand layer at depths from 5 cm to 60 cm ranged from 0.1 to 5.2% (average of 2.8%). Similar values at depths from 5 cm to 1 m at site QH-4 ranged from 6.5% to 18.9% (average of 9.0%) and that at site QH-5 ranged from 7.4% to 26.7% (average of 14%). If the effect of local rainfall is removed, the water content within the sand layer becomes less than 10%. It follows that dry sand layers in the Honglianghe River Basin cannot play a role in protecting the underlying permafrost.

## Conclusions

Desertification cannot play a role in protecting the underlying permafrost of the Qinghai-Tibet Plateau for the following reasons: (1) Permafrost distribution has a strong spatial heterogeneity. Changes in permafrost temperatures are not simply caused by the ground surface being covered with a sand layer; (2) Soil temperatures measured at nine sites in of the Honglianghe River Basin suggest that permafrost temperatures at sites with varying thicknesses of sand cover can be either colder or warmer than those without a sand cover. Local changes in permafrost temperature cannot be solely determined by the thickness of the sand layer or sand cover; (3) The annual heat budget within a sand layer mainly depends on its moisture content. A high moisture content can better preserve underlying permafrost when heat convection due to rainfall infiltration during the summer is disregarded. However, a sand layer generally cannot maintain a high water content because of enhanced rainfall infiltration.
